# Can Targeting Iron Help in Combating Chronic Pseudomonas Infection? A Systematic Review

**DOI:** 10.7759/cureus.13716

**Published:** 2021-03-05

**Authors:** Amena Firoz, Muhammad Haris, Khadija Hussain, Maham Raza, Deepak Verma, Manel Bouchama, Karez S Namiq, Safeera Khan

**Affiliations:** 1 Pediatrics, California Institute of Behavioral Neurosciences & Psychology, Fairfield, USA; 2 Internal Medicine, California Institute of Behavioral Neurosciences & Psychology, Fairfield, USA; 3 Radiology, California Institute of Behavioral Neurosciences & Psychology, Fairfield, USA; 4 Oncology, California Institute of Behavioral Neurosciences & Psychology, Fairfield, USA

**Keywords:** iron metabolism, pseudomonas infections, siderophores, gallium, iron chelators, iron uptake, cystic fibrosis, biofilm formation, cefiderocol, iron

## Abstract

Cystic fibrosis is an autosomal recessive disorder caused by a mutation in genes for cystic fibrosis transmembrane conductance regulator (CFTR) protein. CFTR gene is responsible for the production of sweat, digestive fluids, and mucus, and any mutation in this would lead to the thickening of these secretions. Cystic fibrosis is a multi-organ disorder, but 80% of patients suffer from respiratory problems due to chronic infections most commonly caused by *Pseudomonas aeruginosa (P. aeruginosa)*. Eradication of these infections has become a challenge as* P. aeruginosa* has developed resistance to multiple antibiotics. In several studies, iron has been shown to play an integral role in biofilm formation*, *which is the predominant resistance mechanism used by* P. aeruginosa* to combat antibiotics. The increased iron content in cystic fibrosis patients' sputum samples explains their increased susceptibility to *Pseudomonas* infections. Hence in this review article, we have used the research data available on therapeutic agents that target iron as an adjuvant treatment for chronic *Pseudomonas *infection. We systematically screened three databases using focused words and Medical Subject Headings (MeSH) terms for relevant articles. Further, we applied the inclusion and exclusion criteria and performed a thorough quality appraisal. Thirty shortlisted relevant studies were meticulously reviewed. In our opinion, novel therapeutic approaches targeting iron such as iron chelators, gallium, and cefiderocol have potent anti-biofilm properties. Future studies and clinical trials using these approaches in the management of chronic *Pseudomonas* infection might help in decreasing morbidity and mortality in patients with cystic fibrosis. Exploring these approaches might also help to combat other resistant organisms whose survival is dependent on iron.

## Introduction and background

Cystic fibrosis, first described by Dr. Dorothy Andersen in 1938, has become the most common life-limiting recessive genetic disorder in Caucasians [1]. Approximately 70,000 people live with cystic fibrosis worldwide (more than 30,000 in the USA) [1,2]. One thousand new cases are diagnosed each year, with males and females affected in equal numbers, and more than 75% are diagnosed at two years of age [1,2].

Cystic fibrosis is caused by mutations in the cystic fibrosis transmembrane conductance regulator (CFTR) gene, located on chromosome 7 (7q31.2) [1]. This gene is fundamentally expressed in the airways' epithelium, intestinal tract, pancreas, and sweat glands. It is responsible for regulating the transfer of chloride and sodium across cell membranes, and hence its mutation leads to the thickening of the mucus and other secretions [3]. 

Cystic fibrosis affects multiple organ systems, especially the lungs and pancreas, but intestines, liver, sweat glands, and reproductive organs also are frequently affected [1]. Approximately 80-95% of patients with cystic fibrosis give in to respiratory failure caused by chronic bacterial infection [4]. One of the detrimental opportunistic pathogens that colonize the adult cystic fibrosis lung is* Pseudomonas aeruginosa (P. aeruginosa) *[5]. According to the Cystic fibrosis Foundation Registry Annual Report 2018, 45.3% of cystic fibrosis patients in the US were recorded to have *P. aeruginosa* cultured from their sputum, and 16.9% of them were positive for multidrug-resistant *P. aeruginosa* [2].

**Figure 1:**
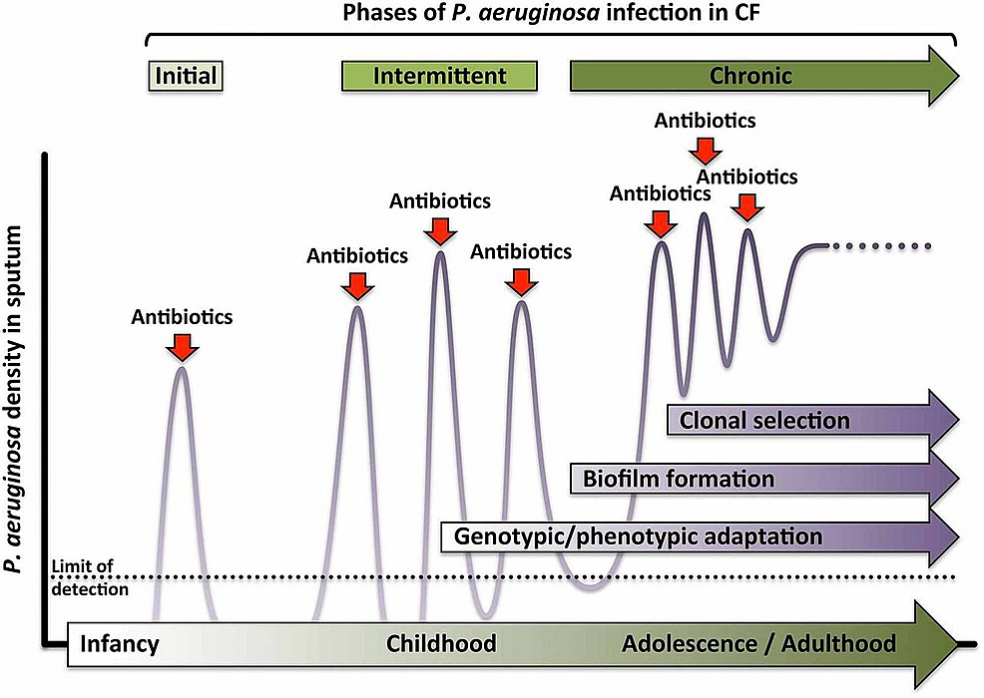
Timeline of Pseudomonas aeruginosa colonization in cystic fibrosis Initial infection with *Pseudomonas aeruginosa* is eradicated with antibiotics. Subsequent intermittent infections cause impairment in bacterial clearance due to genetic adaptation of the bacteria to cystic fibrosis airways. The adaptations form biofilms, production of mucoid coating, and altered expression of virulence factors, leading to chronic infections Reprinted with permission from Nicole M. Bouvier [10]

*P. aeruginosa *combats antibiotic attack with either intrinsic, acquired, or adaptive resistance [6], adaptive resistance being the most pertinent to our review article. It involves biofilm formation, a structured aggregate of bacteria implanted in a self-produced matrix consisting of polysaccharide, protein, and DNA [7]. Biofilms help to endure unpredictable changes in temperature fluctuation and nutrient availability and also serve as a diffusion-barrier to limit antibiotic access to the bacterial cells [8-9]. Figure 1 below illustrates the distinctive time course of* P. aeruginosa* colonization of the cystic fibrosis respiratory tract and resistance mechanisms.

Acute *Pseudomonas* Infections can be eradicated if treated early, but successful therapy is currently unavailable to eliminate a chronic *P. aeruginosa* infection due to the rise of antibiotic resistance. The development of new antibiotics is a laborious process; hence antibiotic adjuvants, agents that act along with a concurrently administered antibiotic, enhancing its action, may offer a novel therapeutic approach to treat antibiotic-resistant* Pseudomonas* infections [11]. One of the targets for therapy currently being investigated is iron, as it has been a critical factor in biofilm formation in* P. aeruginosa* [12]. Regulating microorganismal survival by limiting iron availability may be achieved using agents that target iron uptake pathways and/or iron chelators [13].

While the recommendation of interrupting bacterial nutrition as an antimicrobial strategy was put forward by Louis Pasteur in the 1800s, therapeutic strategies targeting vital bacterial nutrients have been difficult to develop [14,15]. Hence, this systematic review aims to learn about iron metabolism in cystic fibrosis patients, iron uptake mechanisms of *P. aeruginosa*, and new strategies targeting iron to help subdue chronic *Pseudomonas *infections in cystic fibrosis patients.

## Review

Methods

We conducted a systematic review following Preferred Reporting Items for Systematic Reviews and Meta-Analyses (PRISMA) guidelines [16].

Data Source and Strategy

We searched for articles indexed in PubMed, PMC, MEDLINE (National Library of Medicine), and Google Scholar (Alphabet Inc., Mountain View, CA) from December 28 to December 30, 2020. We applied keywords and Medical Subject Headings (MeSH) terms individually and in combination to identify relevant articles. Also, articles referenced in those identified in this search were reviewed for relevance. At the end of our search, we eliminated duplicate articles. Table 1 and Table 2 summarize the search strategy using MeSH terms and keywords.

**Table 1:** Search strategy with MeSH terms MeSH: Medical Subject Headings

MeSH strategy	Search results
("Iron Chelating Agents" [Pharmacological Action]) AND "Pseudomonas Infections/therapy"[Mesh]	24
("Pseudomonas Infections/therapy"[Mesh]) AND "Biofilms/growth and development"[Mesh]	152
("Biofilms/growth and development"[Mesh]) AND "Iron/metabolism"[Mesh]	152
("Iron/metabolism"[Mesh]) AND "Cystic Fibrosis/therapy"[Mesh]	11
("Cystic Fibrosis/etiology"[Mesh]) AND "Pseudomonas Infections/physiopathology"[Mesh]	170
("Iron Chelating Agents/therapeutic use"[Mesh]) AND "Cystic Fibrosis/therapy"[Mesh]	2
("Pseudomonas Infections/etiology"[Mesh]) AND "Biofilms/growth and development"[Mesh]	334
("Pseudomonas Infections/mortality"[Mesh]) AND "Cystic Fibrosis/microbiology"[Mesh]	23
("Pseudomonas aeruginosa/metabolism"[Mesh]) AND "Iron/metabolism"[Mesh]	307

**Table 2:** Search strategy with keywords

Keywords in combination	Search results
iron metabolism in cystic fibrosis	307
Pseudomonas infection and iron uptake	108
iron chelators in cystic fibrosis	103
Pseudomonas biofilm formation and iron	259
Siderophores in Pseudomonas infection	353
iron chelators in Pseudomonas infection	195

Study Selection and Eligibility Criteria

Once we identified the relevant articles, we screened their titles and read through the available abstract and full text of pertinent studies. We included peer-reviewed articles that were published in the English language in the last 15 years. We focused on chronic *Pseudomonas* infection without any age or gender discrimination. The entire review was done scientifically and within ethical boundaries. 

Risk of Bias Assessment

The quality of included studies was assessed by the following tools:

a) Newcastle-Ottawa checklist - observational/non-randomized controlled trial

b) Scale for the Assessment of Narrative Review Articles (SANRA) checklist - traditional review articles 

c) Joanna Briggs Institute (JBI) checklist - case reports/case series

Only those articles that satisfied >70% of the checklist quality parameters were included in the review.

Results

Study Identification and Selection Results

**Figure 2:**
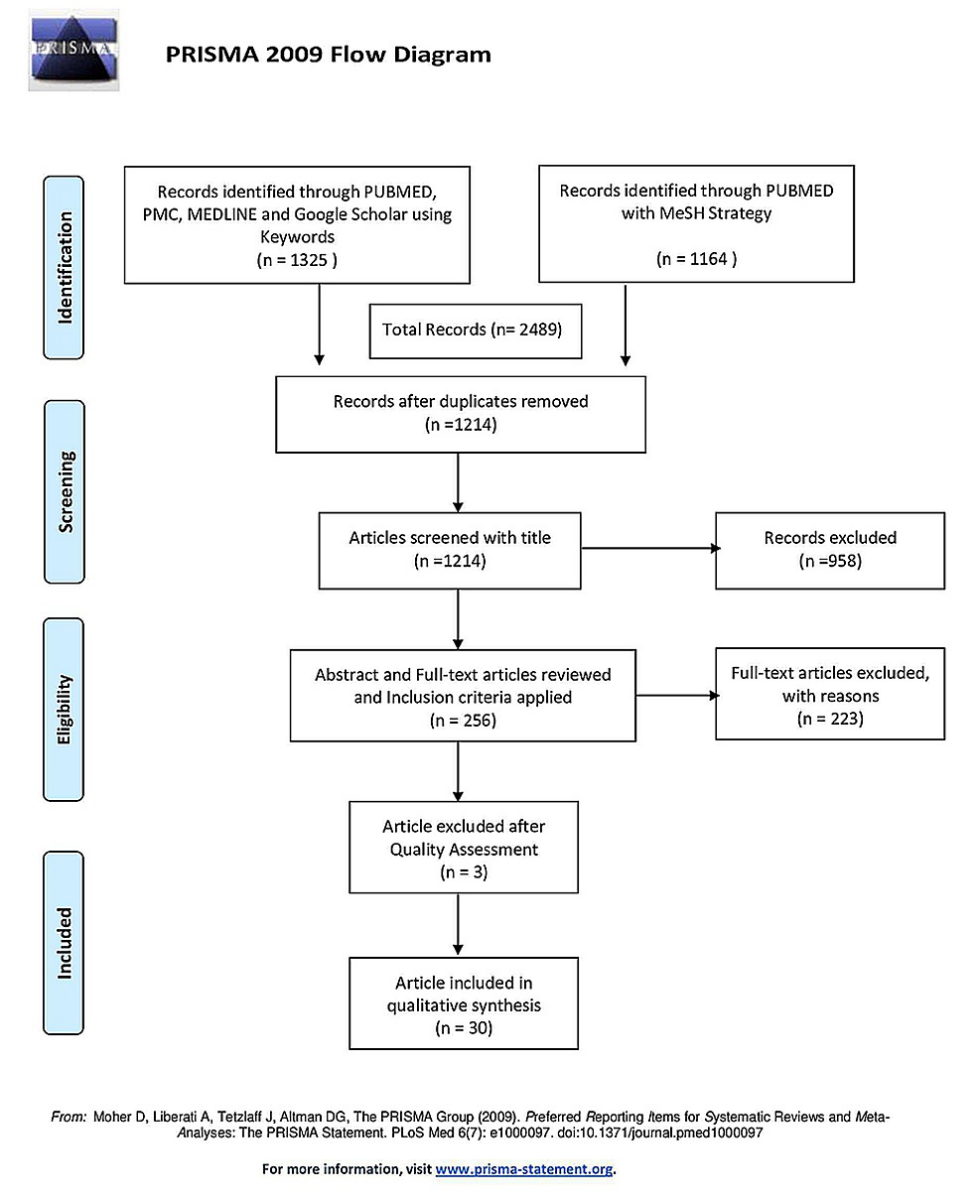
PRISMA flow diagram outlining the search process PRISMA: Preferred Reporting Items for Systematic Reviews and Meta-Analyses

We used four databases to collect relevant articles: PubMed, PMC, MEDLINE, and Google Scholar. Our initial search yielded 2,489 articles: 1,325 identified with keywords, and 1,164 using the MeSH Strategy. After the duplicates were removed (n=1,275), 1,214 articles were screened by title for relevance, following which 985 non-relevant articles were excluded. Abstracts and full text of the 256 relevant articles that remained were thoroughly read, and 223 of them were excluded after inclusion criteria were applied. Lastly, after meticulous quality assessment, three articles were further excluded, and finally, 30 articles were included in the review. Figure 2 depicts the search process in the form of a PRISMA flow diagram.

Quality Assessment

We carried out a rigorous quality assessment for the 33 finalized articles by using various tools like the Newcastle-Ottawa checklist (n=3), the SANRA checklist (n=16), and the JBI checklist (n=12). Two articles were excluded due to unavailable full-text articles for assessment and one article was excluded as it did not satisfy our cut-off (>70%). Therefore, we were finally left with 30 articles to include in our review.

Study Characteristics 

The finalized articles were all peer-reviewed articles published in the last 15 years with full texts freely available. All articles were in English, and articles in other languages were excluded due to unavailable English translations. Human and in vitro studies were included. The condition investigated was Chronic *Pseudomonas* infection. Hence, cystic fibrosis subjects with chronic infection or cells from cystic fibrosis patients' airways or different strains of *P. aeruginosa *were studied in reviewed articles; 50% of the reviewed articles were traditional reviews, and the remaining 50% included observational studies, in vitro studies, and a few case series.

Discussion

We studied 30 previously published articles to understand the importance of iron in chronic *Pseudomonas *infections, mainly concerning cystic fibrosis patients, in order to gain an updated understanding of the novel therapeutics developed against the same.

Iron Metabolism in Cystic Fibrosis Patients 

The normal airway is usually iron-depleted, whereas, in cystic fibrosis, tissue damage due to chronic inflammation and infection increases the airway's iron content [12]. A study conducted by Moreau-Marquis et al. showed that the expression of the most common mutation of CFTR-ΔF508-CFTR mutation in airway cells increases iron release, which enhances biofilm formation, thereby increasing resistance of* P. aeruginosa* to tobramycin (Tb); therefore, the amount of Tb required to eliminate* P. aeruginosa *biofilms on airway cells is 10 times more than the amount achievable in the lungs of cystic fibrosis patients [12].

A similar study conducted by Reid et al. showed that increased sputum iron content in stable cystic fibrosis patients had a direct relationship with* P. aeruginosa *quantitative load, which indicates that iron is a vital factor in its growth and survival [17]. Also, some patients with cystic fibrosis who had no detectable* P. aeruginosa* presence had increased sputum iron and ferritin levels than normal controls, implying increased iron may be an early event that precedes* P. aeruginosa *infection [17]. Apart from enhancing the growth of *P. aeruginosa* in cystic fibrosis patients, elevated iron levels also hamper the host's innate immune system by impairing neutrophil function and rapidly saturating the binding capacities of lactoferrin and transferrin, thereby neutralizing their antimicrobial effects [18-20].

Iron Uptake Pathways by Pseudomonas Aeruginosa

**Figure 3:**
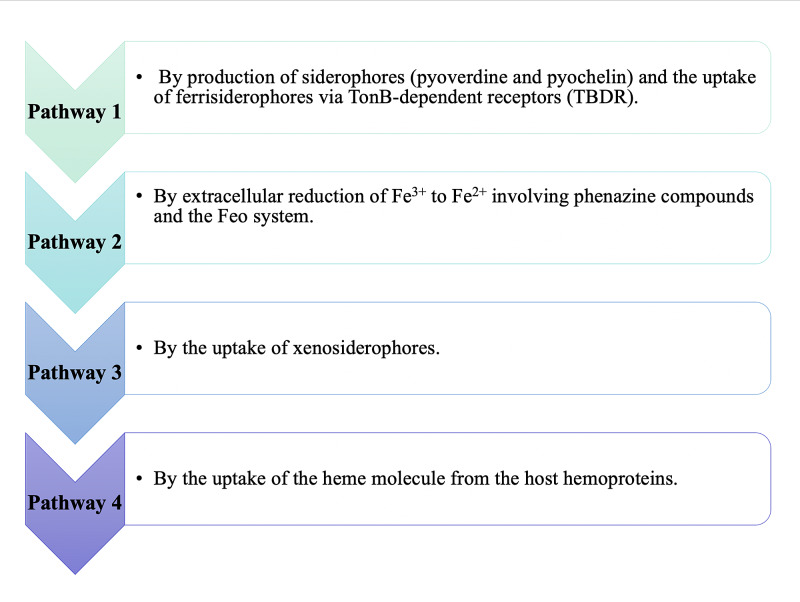
Iron uptake pathways by Pseudomonas aeruginosa Fe2+: ferrous ion; Fe3+: ferric ion

Iron is essential for almost all pathogens; it is also required in numerous vital processes, including DNA synthesis, electron transport, and defense mechanisms [21]. Hence, to cope with iron limitation, bacterial pathogens develop various iron-acquiring pathways that have been described in a study conducted by Cornelis et al., and Figure 3 highlights these pathways [22].

*P. aeruginosa* adapts to different pathways depending on the type of infection (acute vs. chronic); Nguyen et al., in their study, showed that iron is scavenged during acute infections by siderophore pyoverdine, and during prolonged infection, heme is an important source of iron [23]. Apart from the type of infection, iron concentration in the environment also plays a role in the type of endogenous siderophore used for iron acquisition; for example, Banin et al. in their study, state that siderophore pyoverdine is active at low external iron concentration and pyochelin at higher iron levels as pyoverdine has a stronger affinity for iron [24].

*Biofilm Formation - A Survival Mechanism*****

Chronic *Pseudomonas* infections prevail as the bacteria have developed mechanisms to retaliate against antibiotics; Pang et al., in their review, classify these mechanisms into intrinsic, acquired, and adaptive resistance [6]. Intrinsic resistance includes low outer membrane permeability, expression of an efflux pump, and formation of antibiotic-inactivating enzymes [6]. The acquired resistance can be obtained through resistance genes or mutational changes [25]. Lastly, adaptive resistance requires the formation of biofilms that limit antibiotic access to bacteria [7]. Multidrug-tolerant persister cells that form in a biofilm also contribute to recurrent infections. They can withstand an antibiotic attack and regenerate the biofilm once the antibiotic is stopped [26,27].

**Figure 4:**
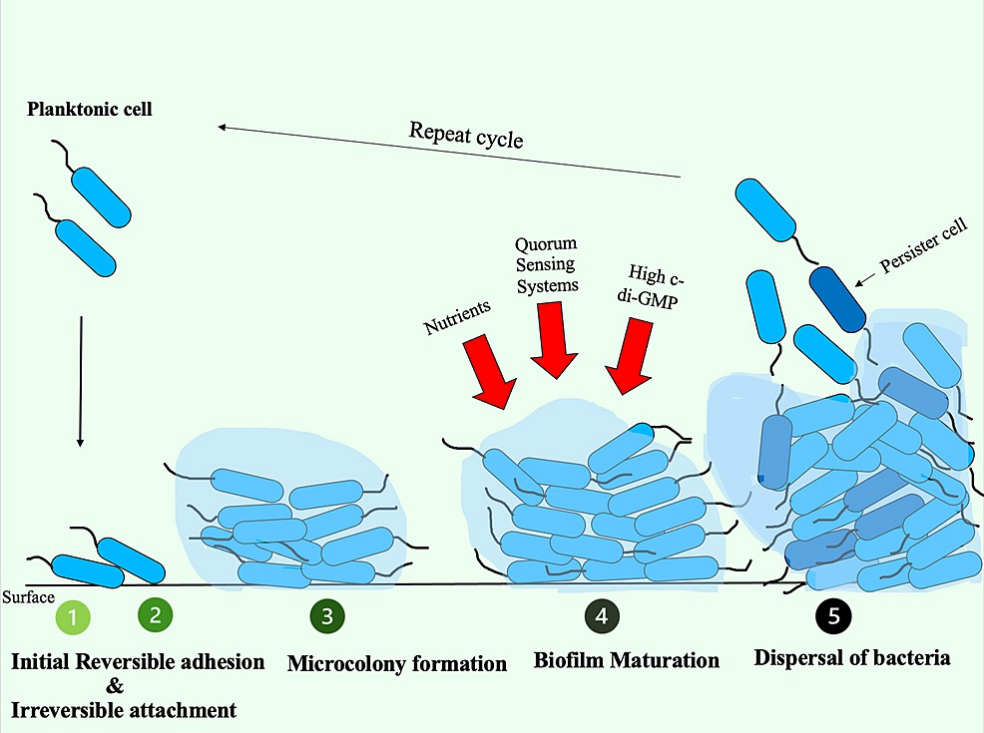
Five steps in biofilm formation c-di-GMP: cyclic diguanylate

In 2005, Banin et al. conducted a study in which they described biofilm formation in terms of four important steps: (I) attachment, (II) microcolony formation, (III) maturation of biofilm, and (IV) dispersal, whereas a review article published by Thi et al. in 2020 split the first step into (attachment) initial reversible adhesion and irreversible attachment, thereby making it five steps in biofilm formation [24,9]. Figure 4 is a pictorial representation of the same.

Biofilm formation is a coordinated effort by individual cells to adapt to cell density changes and environmental stress through an interconnected signaling pathway called quorum sensing (QS). There are four clear pathways in the QS circuits of *P. aeruginosa*, namely Las, Rhl, PQS, and IQS, of which PQS is triggered by variation in iron concentration [9]. In their study, Yang et al. presented evidence that during low iron concentration, biofilm development occurs via stimulation of the PQS genes and the formation of extracellular DNA. In contrast, the inverse process occurs during high iron levels [28].

*Novel Therapeutics Targeting Iron*****

**Figure 5:**
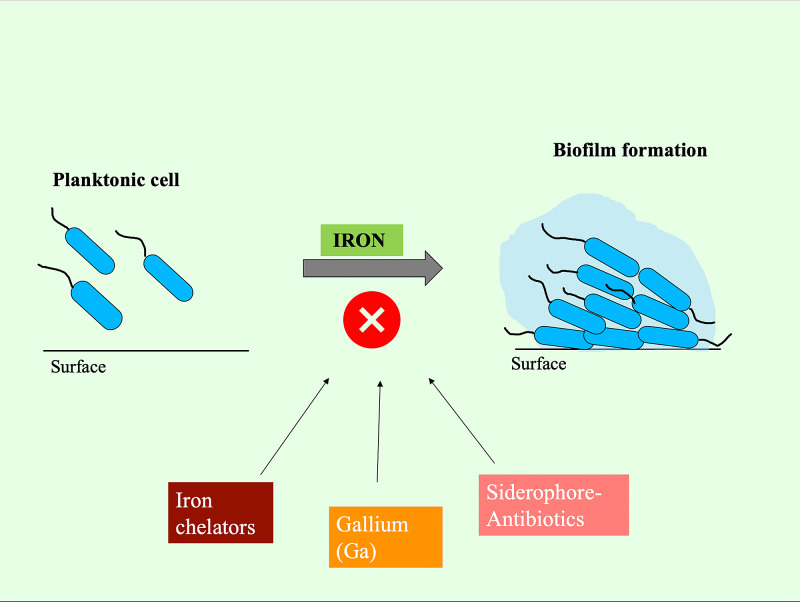
Novel therapeutics targeting iron: iron chelators, gallium, and siderophore-antibiotics

A total of 53% of children diagnosed with cystic fibrosis through newborn screening developed *Pseudomonas* infection by five years of age. These chronic pulmonary infections cause a rapid decline in lung function and increase mortality [29,30]. It has been speculated that a defect in the CFTR gene may be making patients more susceptible to *Pseudomonas* infections by increasing adherence or reducing clearance of the bacterial cells [31,32]. The innate ability of *P. aeruginosa* to resist antibiotics and have different phenotypic variations has made its eradication challenging. Still, over the last few years, new therapeutics targeting its nutrition and chemical signaling pathways are on the rise. Multiple studies in the past have shown that the availability of cytosolic iron greatly influences biofilm formation. For example, in a study conducted by Soldano et al., blocking the BfrB-Bfd complex, which helps mobilize and reduce ferric ions to ferrous ions, created an iron deprivation state in which bacterial cells poorly developed biofilms despite iron-sufficient culture conditions [33]. Hence the therapeutic strategies we discussed in this review, are iron chelators, gallium, and siderophore-antibiotics, as shown in Figure 5.

Exogenous iron-chelating agents: our human body can combat any type of invasion through its innate defense mechanism. Lactoferrin is an endogenous iron chelator that at a sub-bactericidal concentration can identify and block biofilm formation by inducing twitching, a type of motility that promotes a planktonic bacterial cell state rather than forming a cluster or biofilm [34]. However, it has been shown that in cystic fibrosis airways, the action of lactoferrin is overpowered by siderophores as they have a stronger affinity toward iron, and also increased proteolysis of lactoferrin impairs its ability to cease biofilm formation [35,36]. Taking cues from this defense mechanism, scientists have tried to study the role of exogenous iron chelators like the FDA-approved deferoxamine (DFO) and deferasirox (DSX) in the inhibition of biofilm formation. Several laboratory studies have shown that using iron chelators as an adjuvant to antibiotics has a more preferable anti-biofilm effect than antibiotic use alone. For example, a study conducted by Mettrick et al. has shown that N, N'-bis (2-hydroxybenzyl) ethylenediamine-N, N'-diacetic acid (HBED), a synthetic iron chelator in combination with colistin successfully inhibited biofilm formation in aerobic and anaerobic condition [37]. In a similar study conducted by Moreau-Marquis et al., the combination therapy of tobramycin and iron chelator reduced the formation of biofilms by 90% [38]. Thi et al., in their review, highlighted cytotoxicity as one of the drawbacks of iron chelator therapy, which is a concern well addressed in a review by Aali et al., which suggests the use of inhalation route of administration, which not only reduces the potential systemic side effects of the iron chelators but also increases the local action of the drug on the lungs [9,39].

Gallium: the use of metal gallium to derange iron metabolism in organisms that thrive on it for various reasons has been contemplated for decades. Gallium has a similar ionic radius as iron, and hence can be easily mistaken for iron by bacterial uptake systems, and its incorporation into iron-containing protein interrupts its function [40,41]. It also has a noticeable effect on iron metabolism whereby it promotes iron starvation by repression of heme/hemoglobin uptake receptor, pyoverdine synthesis, and pyoverdine receptors [42]. Kaneko et al., in their study, show that gallium inhibits* P. aeruginosa* growth and biofilm formation in vitro by interfering with bacterial uptake and signaling of iron [43]. A similar study conducted by Goss et al. elucidates the important characteristics of gallium that can help impede the growth of *P. aeruginosa*, like inhibiting *P. aeruginosa* ribonucleotide reductase activity, increasing bacterial sensitivity to oxidants, and synergistic activity with some antibiotics [15]. We also learn that gallium resistance develops at a slow rate, and unlike lactoferrin, it does not hamper the killing capacity of human macrophages [15]. Gallium is known to be a nephrotoxic drug. Still, when administered as a slow IV infusion to patients who are encouraged to consume two liters of water during the infusion period, on the one hand, it does not affect kidney function, electrolyte levels, and blood counts, and on the other, improves lung function in cystic fibrosis patients [15].

Cefiderocol-siderophore antibiotics: cefiderocol is an injectable siderophore cephalosporin that is structurally similar to third and fourth-generation cephalosporins. Cefiderocol, like other cephalosporins, inhibits cell wall synthesis by binding to penicillin-binding proteins in the cell wall of Gram-negative bacteria [44]. However, its enhanced stability to β-lactamases and the ability to enter bacterial periplasmic space by utilizing its siderophore-like property make it distinctive [44]. Cefiderocol is considered more potent than ceftazidime-avibactam and meropenem against all resistant forms of *P. aeruginosa*; hence, clinical trials are being conducted to get it approved for clinical use [44].

Table 3 summarizes the studies we reviewed from which we learned about these new therapeutic strategies.

**Table 3:** Studies describing the novel therapeutics targeting iron

Authors	Year	Type of study	Purpose of the study	Relevant results/conclusion
Thi et al. [9]	2020	Traditional review	Investigate current diagnostics of *P. aeruginosa* infections and the treatment of P*. aeruginosa* biofilms	Iron chelators were discussed as a therapeutic strategy where bactericidal, biofilm prevention, and low risk of resistance development stated advantages and cytotoxicity as limitations
Moreau-Marquis et al. [12]	2008	In vitro study	Study the antibiotic resistance of* P. aeruginosa* biofilms grown on airway cells and variation of biofilm formation on ΔF508-CFTR airway cells	The amount of tobramycin required to eliminate* P. aeruginosa* biofilms on airway cells is drastically higher than the amount present in the lungs of cystic fibrosis patients. Airway cells expressing ΔF508-CFTR increased *P. aeruginosa *biofilm formation due to the increased presence of iron
Goss et al. [15]	2018	In vitro study	Understand in-depth about gallium and conduct a human trial to test its efficacy in patients with cystic fibrosis	Gallium works by inhibiting ribonucleotide reductase activity in* P. aeruginosa*. Phase Ib clinical trial showed that it improved lung function and was relatively safe when used in cystic fibrosis patients
Reid et al. [17]	2007	Observational Study	Investigate whether the increase in iron within the cystic fibrosis lung microenvironment has a role in promoting bacterial replication or whether it simply represents an inflammatory state	A direct relationship was found between sputum iron and* P. aeruginosa *growth in clinically stable patients, but not in samples obtained during an acute exacerbation
Nguyen et al. [23]	2014	In vitro study	Understand the changes in iron homeostasis during chronic infections of the cystic fibrosis lung by* P. aeruginosa*	Pyoverdine production reduces with the disease's progression, leading to the evolution of iron regulatory pathways like the heme pathway, which maintains iron homeostasis in the absence of this other iron uptake system
Banin et al. [24]	2005	In vitro study	Examine the impact of mutations in iron acquisition-signaling genes on biofilm morphology	*P. aeruginosa* acquires iron and supports normal biofilm formation by using the siderophores pyoverdine or pyochelin according to iron's availability. In the absence of iron uptake, *P. aeruginosa f*orms atypical biofilms
Yang et al. [28]	2007	In vitro study	Provide information if the variation in the availability of iron affects* P. aeruginosa *DNA release and biofilm formation	Iron at low concentrations promotes* P. aeruginosa *biofilm development via the up-regulation of the PQS genes and the formation of extracellular DNA, and at high levels suppresses biofilm development
Soldano et al. [33]	2020	In vitro study	Understand if a halt in the BfrB-Bfd interaction in *P. aeruginosa *would impair biofilm development	Iron starvation conditions can be established irrespective of the iron concentration in the environment by blocking the BfrB-Bfd complex in *P. aeruginosa,* which negatively affects biofilm formation
Mettrick et al. [37]	2020	Observational Study	Study the biofilm-inhibiting effects of the hexadentate synthetic ferric iron chelator, N, N’-bis (2-hydroxybenzyl) ethylenediamine-N, N’-diacetic acid (HBED), on laboratory and clinical strains of* P. aeruginosa*	HBED and colistin, when used together, can inhibit biofilm formation in both aerobic and anaerobic conditions
Moreau-Marquis et al. [38]	2009	In vitro study	Investigate if iron chelators would enhance tobacco use to treat cystic fibrosis lung infections and eliminate* P. aeruginosa *biofilms	The use of the combination of tobramycin and iron chelator drastically lowered the biomass of established biofilms by 90% of the pre-treatment value
Aali et al. [39]	2017	Traditional review	Understand if the administration of iron chelators to reduce the unregulated ROS production by neutrophils could serve as a novel treatment to improve lung inflammation in cystic fibrosis patients	The inhalation route of administration of iron chelators is the most appropriate as it lowers the chelators' systemic effects by accumulating and acting locally in the lungs
Kaneko et al. [43]	2007	In vitro study	Investigate the use of gallium to disrupt bacterial iron metabolism to inhibit biofilm formation in *P. aeruginosa*	Results showed that gallium inhibits *P. aeruginosa*. growth and biofilm formation. It works by interrupting iron metabolism
Zhanel et al. [44]	2019	Traditional review	Review the existing published data for cefiderocol in detail	Cefiderocol acts against the Gram-negative bacilli by inhibiting cell wall synthesis. Its ability to enter via active iron transport systems has enabled it to overcome β-lactam resistance associated with outer membrane permeability mutations in *P. aeruginosa*

Limitations

Iron chelators demonstrated a very potent anti-biofilm effect in many studies. However, studies that investigated its efficacy were all conducted on laboratory strains of *P.aeruginosa, *which may be different from clinical strains. This is a concern as the clinical strain will respond differently to different iron chelators; hence, the combination of iron chelators may prove more effective clinically. The phase 1 clinical trial conducted with FDA-approved intravenous gallium was small, un-blinded, did not have control, and was limited to patients with mild cystic fibrosis lung disease; thus, the safety and effectiveness of gallium need to be established in future randomized studies with more advanced disease.

## Conclusions

In this systematic review, we aimed to collect information on the significance of iron in chronic *Pseudomonas *infection and determine ways to address it. After thoroughly reviewing the studies, we found that iron played an integral role in helping *P. aeruginosa* combat antibiotics by aiding in biofilm formation and it possesses multiple pathways to acquire iron, the most important being siderophores pyoverdine and pyochelin. Therefore, if the iron is targeted, we might be able to treat antibiotic-resistant* Pseudomonas* infection effectively. Many new therapeutic strategies have been developed over the years, the most promising being iron chelators, gallium, and cefiderocol to inhibit biofilm formation. Iron chelators such as deferoxamine, deferasirox, deferiprone, and IV gallium are FDA-approved and should be considered in future randomized clinical trials.

In our opinion, these new therapeutic approaches can increase survival rates in cystic fibrosis patients as the chronic bacterial infection is one of the most frequent causes of respiratory failure and mortality, and we hope our survey will encourage future clinical trials. These approaches can also be useful in infections caused by other resistant organisms thriving on iron, such as* Escherichia coli* and *Staphylococcus aureus.*
